# Resistance of the respiratory system measured with forced oscillation technique (FOT) correlates with bronchial thermoplasty response

**DOI:** 10.1186/s12931-020-1313-6

**Published:** 2020-02-12

**Authors:** Annika W. M. Goorsenberg, Julia N. S. d’Hooghe, Annelies M. Slats, Joost G. van den Aardweg, Jouke T. Annema, Peter I. Bonta

**Affiliations:** 1 Department of Respiratory Medicine. F5-144, Amsterdam UMC, University of Amsterdam, Meibergdreef 9, 1105 AZ Amsterdam, The Netherlands; 2 Department of Respiratory Medicine, Leiden University Medical Center, Leiden, the Netherlands

**Keywords:** Severe asthma, Bronchial thermoplasty, Forced oscillation technique, Respiratory function tests, Small airways

## Abstract

**Background:**

Bronchial Thermoplasty (BT) is an endoscopic treatment for severe asthma using radiofrequency energy to target airway remodeling including smooth muscle. The correlation of pulmonary function tests and BT response are largely unknown. Forced Oscillation Technique (FOT) is an effort-independent technique to assess respiratory resistance (Rrs) by using pressure oscillations including small airways.

**Aim:**

To investigate the effect of BT on pulmonary function, assessed by spirometry, bodyplethysmography and FOT and explore associations between pulmonary function parameters and BT treatment response.

**Methods:**

Severe asthma patients recruited to the TASMA trial were analyzed in this observational cohort study. Spirometry, bodyplethysmography and FOT measurements were performed before and 6 months after BT. Asthma questionnaires (AQLQ/ACQ-6) were used to assess treatment response.

**Results:**

Twenty-four patients were analyzed. AQLQ and ACQ improved significantly 6 months after BT (AQLQ 4.15 (±0.96) to 4.90 (±1.14) and ACQ 2.64 (±0.60) to 2.11 (±1.04), *p* = 0.004 and *p* = 0.02 respectively). Pulmonary function parameters remained stable. Improvement in FEV_1_ correlated with AQLQ change (*r* = 0.45 *p* = 0.03). Lower respiratory resistance (Rrs) at baseline (both 5 Hz and 19 Hz) significantly correlated to AQLQ improvement (*r* = − 0.52 and *r* = − 0.53 respectively, *p* = 0.01 (both)). Borderline significant correlations with ACQ improvement were found (*r* = 0.30 *p* = 0.16 for 5 Hz and *r* = 0.41 *p* = 0.05 for 19 Hz).

**Conclusion:**

Pulmonary function remained stable after BT. Improvement in FEV_1_ correlated with asthma questionnaires improvement including AQLQ. Lower FOT-measured respiratory resistance at baseline was associated with favorable BT response, which might reflect targeting of larger airways with BT.

**Trial registration:**

ClinicalTrials.gov Identifier: NCT02225392; Registered 26 August 2014.

## Introduction

Bronchial Thermoplasty (BT) is an endoscopic treatment for patients with severe asthma. It uses radiofrequency energy delivered to the medium and larger airways to reduce airway smooth muscle (ASM) mass [1–5]. Several studies have shown an improvement in asthma quality of life, asthma control, and a reduction in exacerbations after BT [6–8]. The exact mechanism of action however is still incompletely understood and patient responder profile remains under debate.

Pulmonary function measurements before and after BT have shown various results and correlations with treatment response have not been explored comprehensively. The large clinical trials and long term follow up studies thereafter showed a stable one-second forced expiratory volume (FEV_1_) up to 5 years after BT with only the RISA trial showing an improvement in FEV_1_ 6 months after BT [6–8].

Forced Oscillation Technique (FOT) is an effort-independent technique using various pressure oscillations to assess the relation between flow and pressure in the respiratory system [9]. It has been postulated that FOT is more accurate in detecting small airways disease than conventional tests such as spirometry [10, 11]. Additionally, while with bodyplethysmography the airway resistance is calculated by combining the flow with alveolar pressure, FOT measures the resistance of the entire respiratory system including the surrounding tissue and small airways [12].

This study hypothesized that the BT-induced reduction of ASM in the larger airways influences the mechanical properties of the asthmatic airways. The aims of this trial are (1) to assess the effect of BT on pulmonary function parameters as assessed by spirometry, bodyplethysmography-determined airway resistance and FOT; (2) to evaluate whether pulmonary function parameters are related to BT response.

## Methods

### Subjects

Patients fulfilling the World Health Organization (WHO) and Innovative Medicine Initiative (IMI) criteria for severe refractory asthma and scheduled for BT and pulmonary function tests including FOT between December 2014 and September 2018 were included (Clinical trials.gov NCT02225392) [13, 14]. Ethical approval was provided by the Medical Ethics Committee of the Academic Medical Center Amsterdam (NL45394.018.13) and written informed consent was obtained. Asthma medication remained stable during the study period.

### Bronchial thermoplasty

Patients were treated with BT according to current guidelines using the Alair System (Boston Scientific, USA) [15–17] and under conscious sedation (remifentanil/propofol) [18] or general anesthesia. Prednisolone 50 mg was started 3 days before treatment, on the day itself and 1 day thereafter.

### Methods of measurement

All pulmonary function tests were performed in the morning and conducted by experienced staff according to ERS/ATS standards using Jaeger Masterlab software (Erich Jaeger GmbH, Wurtzburg, Germany). The measurements were performed during two visits: one visit before and one visit 6 months after treatment. During the visits, short acting bronchodilators were stopped for at least 6 hours. Long acting beta agonists (LABA) were continued. Spirometry, bodyplethysmography and FOT measurements were performed both before and after administration of 400 μg salbutamol. FOT was performed in an upright position with the Resmon Pro device using a pseudorandom noise signal (Restech, Italy). The subjects received a nose-clip and patients were instructed to support their cheeks with their hands while breathing tidal for 3 min. This measurement was performed twice and the average was used in the analysis.

### Outcome parameters

The main outcome parameter of this study was the change in pulmonary function assessed by spirometry, bodyplethysmography and FOT. Other outcome parameters were the correlations between baseline and change in pulmonary function parameters and baseline and change in asthma quality of life questionnaires (AQLQ) and asthma control (ACQ-6) [19, 20]. Changes in pulmonary function parameters or asthma questionnaires were defined as post-BT minus pre-BT values. A decrease of 0.5 points on AQLQ and an increase of 0.5 points on ACQ-6 is designated as clinically relevant.

### Statistical analysis

GraphPad Prism version 5.01 (GraphPad Software Inc., San Diego, CA, USA) was used for the analysis. Grouped data were reported as mean with standard deviation or median with interquartile ranges, as appropriate. Within group analyses were performed with paired t-tests or Wilcoxon signed rank tests. Correlation analyses were performed with Spearman’s rho coefficient. *P*-values were two sided and a statistical significance was set at *p* < 0.05.

## Results

### Subjects and clinical outcome

BT and pulmonary function tests including FOT were performed in 26 patients. Two patients were excluded from analyses due to lost to follow up at the 6 months visit. Baseline characteristics of the included 24 patients are shown in Table 1. Due to claustrophobia one patient was excluded from bodyplethysmography analyses. In FOT analysis, one patient was excluded due to extreme coughing and in one other patient only post-bronchodilator measurements were performed. BT significantly improved quality of life and asthma control. AQLQ questionnaires improved from 4.15 (±0.96) to 4.90 (±1.14) (*p* = 0.004) and ACQ questionnaires improved from 2.64 (±0.60) to 2.11 (±1.04) (*p* = 0.02).

**Table 1:** Baseline characteristics

Characteristics	Baseline
No. of patients	24
Sex (males/females)	5/19
Age (y)	44 ± 12
BMI	28.3 ± 4.9
FeNO (ppb)	17.8 (12.6; 45.6)
Total serum IgE (kU/L)	67 (14; 219)
Blood eosinophil count (10^9/^L)	0.15 (0.06; 0.29)
ACQ score	2.64 ± 0.60
AQLQ score	4.15 ± 0.96
Dose of LABA (μg/d salmeterol equivalents)	135 ± 55
Dose of ICS (μg/d fluticasone equivalents)	1174 ± 508
No. of patients on maintenance use of OCS	7
Dose of oral prednisone (mg/d)	12 ± 6
No. of patients on omalizumab	3

Data are presented as numbers, mean (± SD) or median (IQR)

*BMI* body mass index, *FeNO* fractional exhaled nitric oxide, *ACQ* asthma control questionnaire, *AQLQ* asthma quality of life questionnaire, *LABA* long acting bèta-2-agonist, *ICS* inhaled corticosteroids, *OCS* oral corticosteroids

### Pulmonary function measurements

The effect of BT on spirometry and bodyplethysmography parameters are shown in Table 2. FEV_1_ did not significantly change after BT. FVC (% of predicted, pre-bronchodilator) was slightly increased after BT, with a stable FEV_1_ resulting in a reciprocal decrease in FEV_1_/FVC. Additionally, a minimal increase in post bronchodilator airway resistance was found (before BT 0.15 (0.14;0.21) kPa*s/L versus after BT 0.23 (0.16;0.24) kPa*s/L (*p* < 0.05)).

**Table 2:** Pulmonary function parameters before and after Bronchial Thermoplasty treatment

	Parameter	Before BT	After BT	*P*-value
Before bronchodilation	FEV_1_ (% predicted)	88 ± 21	90 ± 21	0.33
	FEF 75 (L/s)	1.04 ± 0.57	0.97 ± 0.70	0.44
	FVC (% predicted)	98 ± 20	102 ± 17	< 0.05
	FEV_1_/FVC	0.74 ± 0.11	0.73 ± 0.12	0.46
	Raw IN (kPa*s/L)	0.30 (0.20;0.42)	0.29 (0.19;0.42)	0.99
	FRC (L)	2.60 (1.97;3.22)	2.63 (2.03;3.08)	0.69
	RV (L)	1.81 ± 0.74	1.80 ± 0.64	0.68
After bronchodilation	FEV_1_ (% predicted)	100 ± 18	100 ± 15	0.72
	FEF 75 (L/s)	1.28 ± 0.67	1.20 ± 0.78	0.37
	FVC (% predicted)	107 ± 15	108 ± 13	0.33
	FEV_1_/FVC	0.79 ± 0.10	0.77 ± 0.10	< 0.05
	FEV_1_ reversibility	11 (3.5;14)	4 (2;15)	0.11
	Raw IN (kPa*s/L)	0.15 (0.14;0.21)	0.23 (0.16;0.24)	< 0.05
	FRC (L)	2.73 (1.90;3.10)	2.52 (2.00;2.91)	0.14
	RV (L)	1.60 ± 0.55	1.65 ± 0.47	0.49

Data are presented as mean (± SD) or median (IQR); *n* = 23 for pre-bronchodilator measurements and *n* = 24 for post-bronchodilator measurements

*FEV*_*1*_ forced expiratory volume in 1 second, *FEF 75* forced expiratory flow at 75% of the FVC, *FVC* forced vital capacity, *Raw* airway resistance, *FRC* functional residual capacity, *RV* residual volume

Total group analyses of FOT measurements did not show a change in respiratory resistance (Rrs) and reactance (Xrs) after BT for both 5 Hz and 19 Hz (Table 3).

**Table 3:** Forced Oscillation Technique parameters before and after Bronchial Thermoplasty treatment

	Parameter	Before BT	After BT	*P*-value
Before bronchodilation	Rrs 5 Hz (cm H2O*s/L)	3.58 (3.21; 4.24)	3.70 (2.88; 4.29)	0.59
	Xrs 5 Hz (cm H2O*s/L)	−1.23 (−1.68; −0.89)	−1.28 (− 1.81; − 0.95)	0.21
	Rrs 19 Hz (cm H2O*s/L)	3.17 (2.85; 3.56)	3.15 (2.80; 3.67)	0.95
	Xrs 19 Hz (cm H2O*s/L)	0.65 (0.09; 0.99)	0.63 (−0.04; 0.80)	0.22
After bronchodilation	Rrs 5 Hz (cm H2O*s/L)	3.06 (2.73; 3.41)	3.22 (2.78; 3.63)	0.08
	Xrs 5 Hz (cm H2O*s/L)	−1.00 (−1.15; −0.77)	−0.92 (− 1.23; − 0.73)	0.28
	Rrs 19 Hz (cm H2O*s/L)	2.90 (2.65; 3.09)	2.97 (2.72; 3.47)	0.26
	Xrs 19 Hz (cm H2O*s/L)	0.80 (0.56; 1.07)	0.87 (0.27; 1.08)	0.21

Data are presented as median (IQR); *n* = 22 for pre-bronchodilator measurements and *n* = 23 for post-bronchodilator measurements

*Rrs* respiratory resistance, *Xrs* reactance

### Correlation analyses

Associations between asthma questionnaires and pulmonary function parameters were explored.

#### Asthma questionnaires and spirometry parameters

**Fig. 1:**
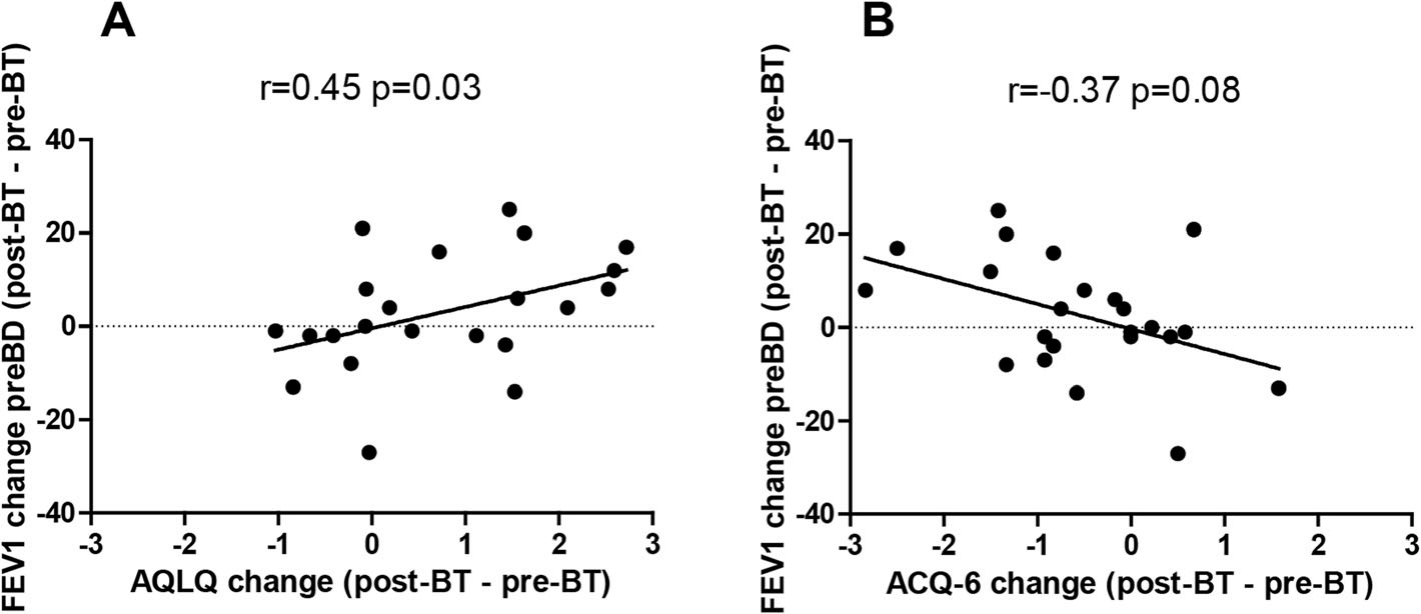
Correlation between asthma questionnaire AQLQ and ACQ-6 changes and pre-BD FEV1(% predicted) change after BT. An improvement in AQLQ (*n* = 22) (**a**) and ACQ (*n* = 23) (**b**) is correlated with post-BT change in FEV1 (%) pre-BD. FEV1, forced expiratory volume in 1 s; BD, bronchodilation; BT, Bronchial Thermoplasty; AQLQ, asthma quality of life questionnaire; ACQ, asthma control questionnaire

No significant correlations were found at baseline before BT between asthma questionnaires and spirometry parameters. After BT, improvements in AQLQ and ACQ showed a correlation with baseline FEV_1_ reversibility (for AQLQ *r* = 0.42 *p* = 0.05 and for ACQ *r* = − 0.45 *p* = 0.03) but not with baseline FEV_1_. Additionally, after BT improvements in asthma questionnaires were correlated with improvements in pre-bronchodilator FEV_1_ (% predicted) (*r* = 0.45 *p* = 0.03 for AQLQ and *r* = − 0.37 *p* = 0.08 for ACQ) (Fig. 1a and b) but not with post-bronchodilator FEV_1_.

#### Asthma questionnaires and bodyplethysmography

Regarding bodyplethysmography, a correlation was found between baseline AQLQ and baseline airway resistance (Raw) (*n* = 23, *r* = 0.56 for both pre-BD and post-BD; *p* < 0.01). Baseline measurements of airway resistance were not correlated with baseline ACQ. No correlations were found between changes in AQLQ and ACQ questionnaires and airway resistance measured with bodyplethysmography.

#### Asthma questionnaires and respiratory resistance

Similar correlations were found for respiratory resistance measured with FOT at both 5hz and 19 Hz. Baseline AQLQ scores showed a significant positive correlation with respiratory resistance at 19 Hz (*r* = 0.67 *p* = 0.0005 for pre-bronchodilator Rrs and *r* = 0.57 *p* = 0.005 for post-bronchodilator Rrs) and a trend between baseline AQLQ and pre-bronchodilator respiratory resistance at 5 Hz (*r* = 0.36; *p* = 0.09). Baseline ACQ scores were not correlated to baseline FOT measurements.

**Fig. 2:**
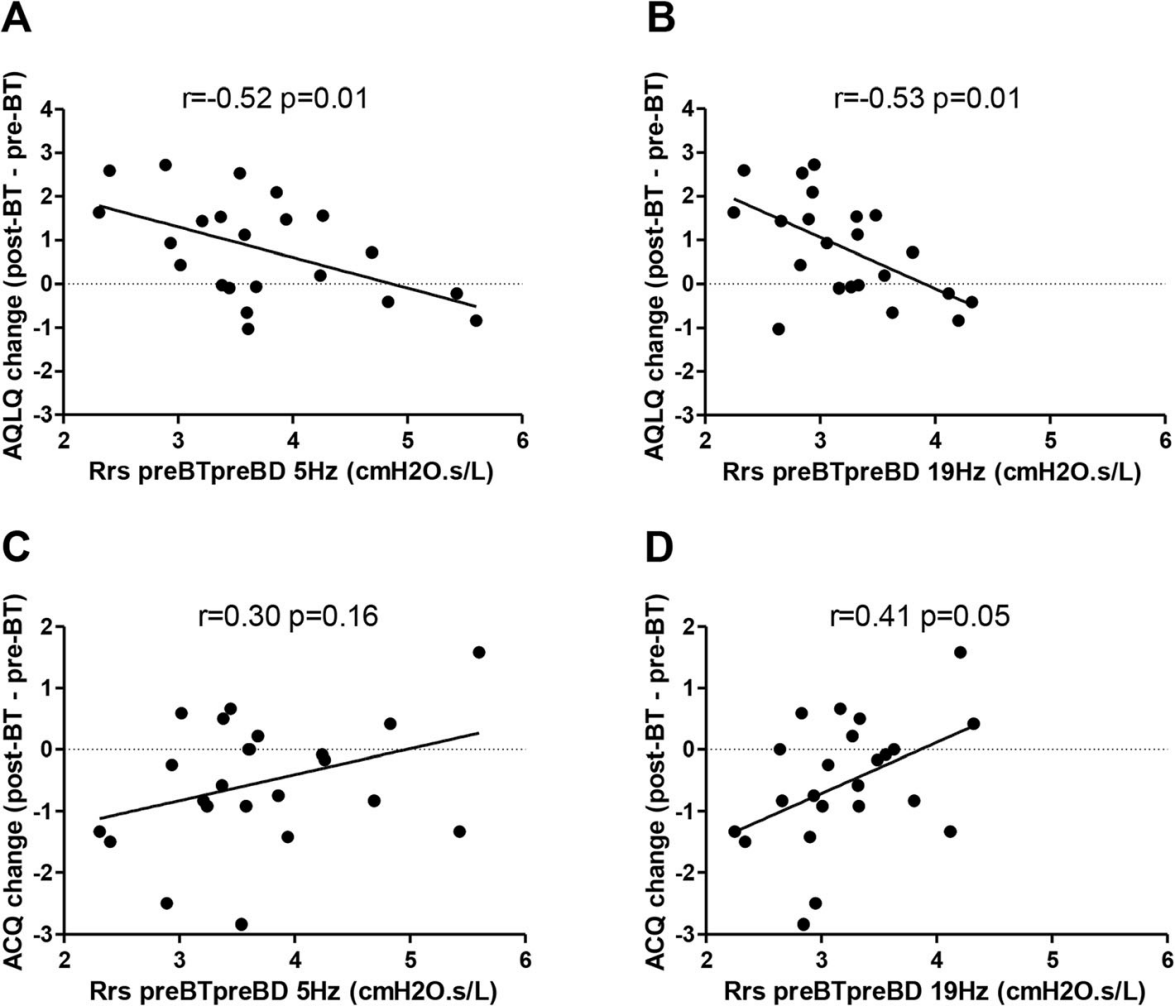
Associations between asthma questionnaire AQLQ and ACQ-6 changes and respiratory resistance measured with FOT at baseline (5 Hz and 19 Hz). A negative correlation was found between AQLQ improvement and baseline respiratory resistance at both 5 Hz (**a**) and 19 Hz (**b**). A positive correlation was seen between ACQ improvement and baseline respiratory resistance at both 5 Hz (**c**) and 19 Hz (**d**). FOT, forced oscillation technique; AQLQ, asthma quality of life questionnaire; ACQ, asthma control questionnaire; Rrs, respiratory resistance in cmH2O.s/L; BT, bronchial thermoplasty; BD, bronchodilation

Next the correlation between changes in asthma questionnaires and baseline respiratory resistance were analyzed. AQLQ improvement was negatively correlated with baseline pre-bronchodilator respiratory resistance (Rrs at 5 Hz *r* = − 0.52 *p* = 0.01; Rrs at 19 Hz *r* = − 0.53 *p* = 0.01) (Fig. 2a-b) and baseline post-bronchodilator respiratory resistance (Rrs at 5hz *r* = − 0.43 *p* = 0.04; Rrs at 19 Hz *r* = − 0.55 *p* = 0.01). A positive trend was found between ACQ improvement and baseline pre-bronchodilator respiratory resistance at both 5 Hz (*r* = 0.30 *p* = 0.16) and 19 Hz (*r* = 0.41 *p* = 0.05) (Fig. 2c-d).

#### Asthma questionnaires and airway reactance

Correlations between asthma questionnaires and reactance measurements were only found for AQLQ and reactance at 5 Hz: baseline AQLQ was negatively correlated with airway reactance (*r* = − 0.42; *p* = 0.05) and improvement of AQLQ was correlated with a higher reactance at baseline (*r* = 0.48; *p* = 0.02). No significant correlation between baseline / change ACQ and airway reactance was found.

## Discussion

This study aimed to investigate the effect of BT on pulmonary function and to explore whether these pulmonary function parameters were associated with BT response. An improvement in asthma control and quality of life was found while overall pulmonary function parameters remained stable. More importantly, this is the first study showing that a low respiratory resistance, measured with FOT, correlated to BT-response. These results can contribute to improved patient selection for BT.

Comparable to previously published larger trials [8, 21], spirometry parameters overall remained stable. A slight increase in pre-bronchodilator FVC (% predicted) and decrease in post-bronchodilator FEV_1_/FVC after BT were found, although significantly different, the clinical relevance of these small differences is questionable. For the first time however, correlations were found between asthma questionnaire (AQLQ and ACQ) changes and FEV_1_ change. Although the differences were small, these data suggest that spirometry might improve after BT as previously shown in the RISA trial [7]. In our study, this improvement in FEV_1_ was only visible in the patients that responded well to BT. This correlation was also explored, but not found in an Australian cohort of severe asthma patients [22]. An explanation can be the difference in baseline characteristics between both cohorts, with a more obstructive asthma phenotype in the Australian cohort compared to the present study (FEV_1_ (% predicted) of 55% compared to 88%).

When taking all patient data into account a significant increase was found after BT in post-bronchodilator bodyplethysmography airway resistance. This increase is mainly caused by one patient, who gained 7 kg during follow-up, which could explain this outcome. Similar to Langton et al. [23] no significant differences in FOT measurements were found after BT in our study. However, a positive correlation was found between airway and respiratory resistance measured with both bodyplethysmography and FOT and AQLQ questionnaires at baseline. For ACQ this correlation was not present. The mechanism underlying this result needs to be further explored.

An important finding of this study is the correlation between improvements on AQLQ and ACQ and respiratory resistance measured with FOT. In this study, patients with a higher respiratory resistance at baseline showed less improvements on both questionnaires after BT compared to patients with a lower resistance. Conventional spirometry and bodyplethysmography-determined airway resistance did not show this correlation. A possible explanation for this difference might be that FOT measures the respiratory resistance of the entire respiratory system, including smaller airways and surrounding tissue. Non-responding patients might be the patients with a higher resistance in surrounding tissue, potentially in the smaller distal airways which are not reached by the BT catheter. Consequently, patients with lower respiratory resistance at baseline might be the patients to select for BT treatment.

An improvement in the respiratory resistance was not observed. Other recently published studies however did show an improvement of ventilation homogeneity after BT [24] and effects of BT on airtrapping parameters with pulmonary function tests [22] and Computed Tomography [25–27] indicating a BT-effect in the peripheral parts of the airways. To measure the resistance in the smaller airways, FOT alone is probably not sufficient. The assessment of small airways disease and/or the effect on the smaller airways of BT might be more accurate when combining multiple techniques together such as CT, FOT and/or impulse oscillometry (IOS) as currently investigated by the Atlantis study group [28].

There are limitations to this study that need to be addressed. The results in this study are part of the TASMA study, a multicenter study, however FOT measurements were only performed in one center. Therefore the present study included patients from one center only. Although single center results, the included group was clinically heterogeneous with allergic, eosinophilic and non-allergic/non-eosinophilic patients included. Additionally, patients were referred to this center from all parts of the Netherlands, thereby decreasing the effect of environmental factors on the outcome. Another limitation is the relatively small number of included patients. Although results need to be confirmed in larger trials, this study does offer important insights that may help to improve patient selection in the future. Strong points of this study are using not only conventional methods to assess lung function parameters but also use FOT, a method known to give a more reliable result on peripheral airway resistance. Also by keeping the medication use stable during follow up, and not start tapering down, which could influence the results, strengthens the observed measurements.

## Conclusion

Pulmonary function parameters, including FOT, remained stable after BT. Correlations were found between FEV_1_ improvement and asthma questionnaires improvement including AQLQ. Additionally, a lower respiratory resistance at baseline, measured with FOT, was associated with a favorable BT-response, which might reflect the main targeting of BT on the larger airways. These results add to understanding the mechanism of action of BT and might contribute to improved patient selection for this treatment.

## Data Availability

The datasets used and/or analysed during the current study are available from the corresponding author on reasonable request.

## Abbreviations

ACQ: Asthma control questionnaire
AQLQ: Asthma quality of life questionnaire
ASM: Airway smooth muscle
BT: Bronchial thermoplasty
CT: Computed tomography
FEV1: Forced expiratory volume in 1 s
FOT: Forced oscillation technique
FVC: Forced vital capacity
IMI: Innovative medicine initiative
IQR: Interquartile range
Rrs: Respiratory resistance
WHO: World health organization
Xrs: Reactance
